# Age-Related Macular Degeneration: Advances in Management and Diagnosis

**DOI:** 10.3390/jcm4020343

**Published:** 2015-02-12

**Authors:** Yoshihiro Yonekawa, Joan W. Miller, Ivana K. Kim

**Affiliations:** Retina Service, Department of Ophthalmology, Massachusetts Eye and Ear Infirmary, Harvard Medical School, Boston, MA 02114, USA; E-Mails: Yoshihiro_Yonekawa@meei.harvard.edu (Y.Y.); joan_miller@meei.harvard.edu (J.W.M.)

**Keywords:** age-related macular degeneration, choroidal neovascularization, fundus autofluorescence, fluorescein angiography, optical coherence tomography, retina, vascular endothelial growth factor, visual impairment

## Abstract

Age-related macular degeneration (AMD) is the most common cause of irreversible visual impairment in older populations in industrialized nations. AMD is a late-onset deterioration of photoreceptors and retinal pigment epithelium in the central retina caused by various environmental and genetic factors. Great strides in our understanding of AMD pathogenesis have been made in the past several decades, which have translated into revolutionary therapeutic agents in recent years. In this review, we describe the clinical and pathologic features of AMD and present an overview of current diagnosis and treatment strategies.

## 1. Introduction

Age-related macular degeneration (AMD) is the most common cause of visual impairment and blindness in the elderly in industrialized nations [1]. In 2000, more than nine million individuals were estimated to have AMD in the United States [2]. Its prevalence is predicted to double by 2020 [3]. AMD is classified into two main forms: non-neovascular (also known as “dry” or “nonexudative”) or neovascular (also known as “wet” or “exudative”). The clinical hallmark of non-neovascular AMD is drusen, which are yellowish deposits at the level of the retinal pigment epithelium (RPE) which lies just under the neurosensory retina. Focal RPE hyperpigmentation and atrophy can also be seen. “Geographic atrophy” is the advanced stage of non-neovascular AMD, where areas of atrophy become confluent and cause visual loss. Neovascular AMD is also an advanced manifestation of AMD, characterized by choroidal neovascularization (CNV)—abnormal blood vessels typically arising in the choriocapillaris and often invading the subretinal space. Only about 10% of patients with AMD have the neovascular form, but it previously caused 90% of blindness related to AMD [4]. However, the recent development of anti-vascular endothelial growth factor (VEGF) agents has revolutionized therapy for this condition, and vision loss can now be avoided in over 90% of patients with neovascular AMD, with about one-third of patients experiencing significant improvements in visual acuity [1]. 

## 2. Risk Factors for AMD

The unequivocal risk factor for any stage of AMD is old age. Pooled data from seven population-based studies showed that the prevalence of geographic atrophy in the United States was 0.3% in 60–64 year olds, 0.5% in 65–69 year olds, 0.9% in 70–74 year olds, 1.8% in 75–79 year olds, and 6.9% in those 80 or older [2]. The respective rates for neovascular disease were 0.4%, 0.6%, 1.2%, 2.2%, and 8.2%. Therefore, as life expectancy improves with advances in medicine and public health, the number of patients affected by AMD is also likely to increase.

The 1988–1994 National Health and Nutrition Examination Survey (NHANES) showed that women had a higher prevalence of AMD than men regardless of race and age [5]. However, this difference was not observed in the more recent 2005–2008 NHANES [6]. While some studies have implicated female gender as an independent risk factor [7,8,9], some have not [2,10,11,12], and some have shown the opposite [13]. The longer life expectancies of women may confound some evaluations of gender and AMD risk.

Advanced AMD is seen more frequently in Caucasian patients. In the Baltimore Eye Study, 30% of bilateral blindness in Caucasians was caused by AMD, compared to 0% in African Americans [14]. Pooled data from the Baltimore Eye Study, Barbados Eye Study, and the Salisbury Eye Evaluation Project also showed that the exponential increase of advanced AMD later in life seen in Caucasians is not observed in African Americans [2]. For example, at 80 years or older, the prevalence of neovascular AMD in Caucasians was 11.1% for women and 8.3% for men, compared to 1.8% and 0.9%, respectively, in African Americans. Similar differences are seen for geographic atrophy.

Smoking is perhaps the only well-established risk factor that is modifiable. The Age-Related Eye Disease Study (AREDS) showed that the risk for neovascular AMD was doubled for participants who had ever smoked [7]. The Physicians’ Health Study showed that current male smokers had double the risk of developing AMD [15], and the Nurse’s Health Study also described similar findings for women [16].

Patients with AMD are likely to have systemic co-morbidities. Most can be attributed to old age as a confounding factor. Numerous cross-sectional studies have investigated independent systemic risk factors for AMD, with conflicting results for most, if not all, factors studied. Hypertension appears to be the most consistently implicated in epidemiological studies [17,18,19,20,21], but antihypertensive medication has not been shown to decrease the risk of AMD [22,23]. Most studies have shown that cerebrovascular disease has no effect on AMD risk [12,18,24,25,26]. Data regarding the association between AMD and atherosclerosis [26,27] as well as dyslipidemia [7,28,29,30] are also inconsistent. The difficulties in establishing definite associations likely stem from the heterogeneity in definitions for both AMD and the various systemic conditions investigated, which also makes comparisons between studies challenging. However, AMD patients are advised to continue efforts to optimize systemic cardiovascular health—from inflammation to lipid metabolism and oxidative damage, there are many common pathophysiologic mechanisms between AMD and cardiovascular disease.

Numerous large studies have confirmed the association between genetic variation at two major loci on chromosome 1 and chromosome 10 and AMD. Polymorphisms in complement factor H in the 1q32 region have been associated with increased risk of all forms of AMD, pointing to the complement pathway and mechanisms related to immunity/inflammation as important factors in AMD pathogenesis. Similarly, variation in the *ARMS2* gene on 10q26 is also associated with increased AMD risk, confirming the role of extracellular matrix regulation and oxidative stress in this disease [1].

## 3. Clinical Features, Histopathology, and Imaging

**Figure 1:**
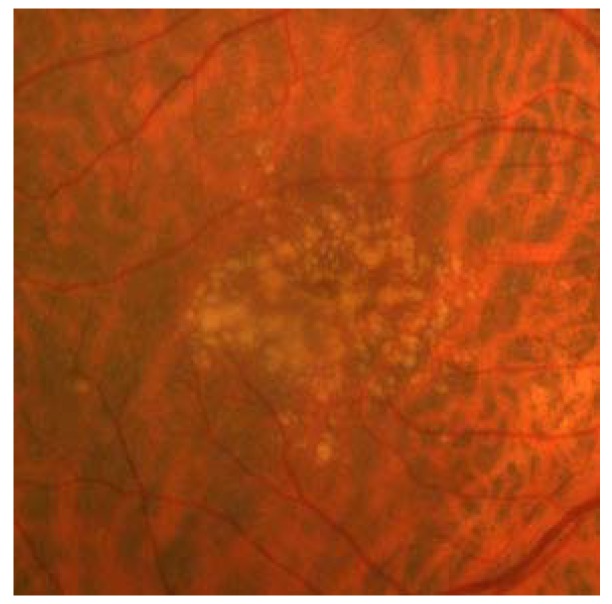
Drusen in non-neovascular age-related macular degeneration appear as round, yellow deposits deep in the retina during fundus examination.

The RPE is susceptible to change with aging because it is a non-replicating tissue that, throughout its lifespan, continually engulfs photoreceptor discs and surrounding cellular contents. Lipofuscin is the result of undigested material that increases with aging and accumulates in the RPE [31,32,33]. Lipoproteins and amyloid-beta also start to accumulate under the RPE; these deposits are called basal laminar deposits (BLamD). Focal, early forms of BLamD are considered a part of normal aging, and usually cannot be seen clinically during fundus examination [1]. However in AMD, the BLamD develop layers of thick hyalinized material and clinically appear as pigmentary changes [34]. AMD is also characterized by basal linear deposits (BLinD), which are located deeper than BLamD at the level of Bruch’s membrane [1]. The clinical correlation of confluent areas with BLinD is soft drusen, the hallmark of AMD (Figure 1). On optical coherence tomography (OCT), a noninvasive imaging technology that provides high-resolution cross-sectional views of retinal tissue, drusen appear as focal RPE deformations. OCT software has analytical features, such as automated segmentation of retinal layers, which allows calculations of the volume of drusen and facilitates longitudinal comparisons of drusen accumulation.

Eyes with early AMD develop BLinD and thin continuous layers of early BLamD, but these lesions are often not detected clinically. From here, there appears to be two pathways of progression. If there is excess production of BLinD, this manifests as intermediate-to-large soft drusen with or without pigmentary changes. The soft drusen then can lead to drusen-related geographic atrophy and/or development of CNV. An alternative pathway is often seen in older individuals and is characterized by consistently low production of BLinD. The fundus initially appears clinically normal, but gradually develops pigmentary changes and finally geographic atrophy without significant drusen. 

## 4. Geographic Atrophy

**Figure 2:**
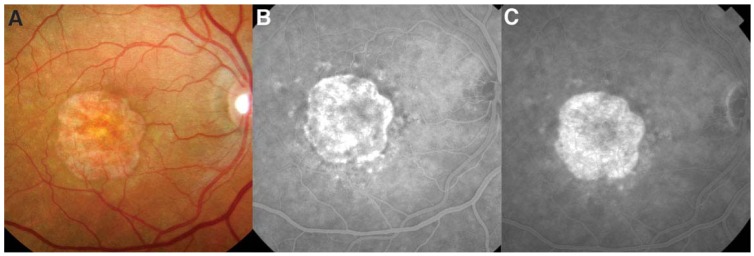
Geographic atrophy is an advanced form of non-neovascular age-related macular degeneration. There is sharply demarcated confluent atrophy of the retinal pigment epithelium and overlying outer retina, with a view of the underlying choroidal vasculature (**A**); on fluorescein angiography, there is a “window defect” during the early frames with transmission of choroidal fluorescence (**B**); which does not leak in later frames (**C**).

During fundus examination, geographic atrophy is seen as sharply delineated areas of RPE atrophy, which clinically appear as areas of hypopigmentation with prominent views of the underlying choroidal vessels (Figure 2). Most geographic atrophy develops in areas of regressed large drusen [35], but may also occur independent of drusen, typically in areas of prior pigmentary changes suggesting RPE dysfunction. On fluorescein angiography, geographic atrophy appears as well-circumscribed areas of hyperfluorescence due to increased transmission from the underlying choroidal vasculature. On OCT, areas of geographic atrophy are characterized by loss of the external limiting membrane, ellipsoid zone, and the RPE-Bruch’s membrane complex. Fundus autofluorescence (FAF) is an accurate, noninvasive method for monitoring the progression of atrophy. The atrophic areas appear as sharply demarcated areas of lost autofluorescence, and hyperautofluorescence of the borders of the lesions is predictive of subsequent progression in those areas [36,37]. The surface area of lost autofluorescence can be measured, which allows quantitation of geographic atrophy growth. FAF is useful in phenotyping drusenoid and atrophic changes in eyes with AMD, as well as in distinguishing AMD from mimicking retinal degenerations such as Stargardt disease and central areolar choroidal dystrophy.

## 5. Neovascular AMD

**Figure 3:**
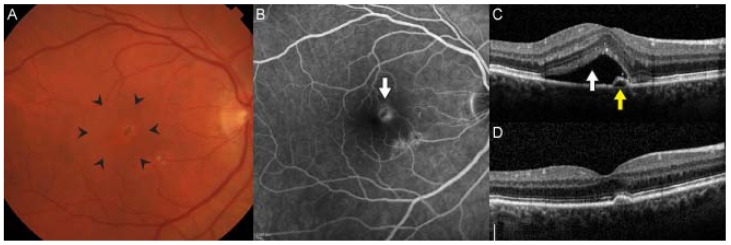
Choroidal neovascularization is the hallmark of neovascular age-related macular degeneration. There is often thickening or elevation of the retina seen clinically through stereoscopic biomicroscopy (area within arrow heads) (**A**); On fluorescein angiography, neovascular membranes appear as hyperfluorescent lesions deep in the retina (arrow) that leak over time (**B**); Spectral-domain optical coherence tomography allows detailed cross-sectional imaging of retinal anatomy. In this patient, there was subretinal fluid (white arrow), and a small adjacent pigment epithelial detachment. Visual acuity was 20/32 (**C**); After 3 monthly intravitreal injections of ranibizumab, the fluid resolved, and visual acuity improved to 20/20 (**D**).

**Figure 4:**
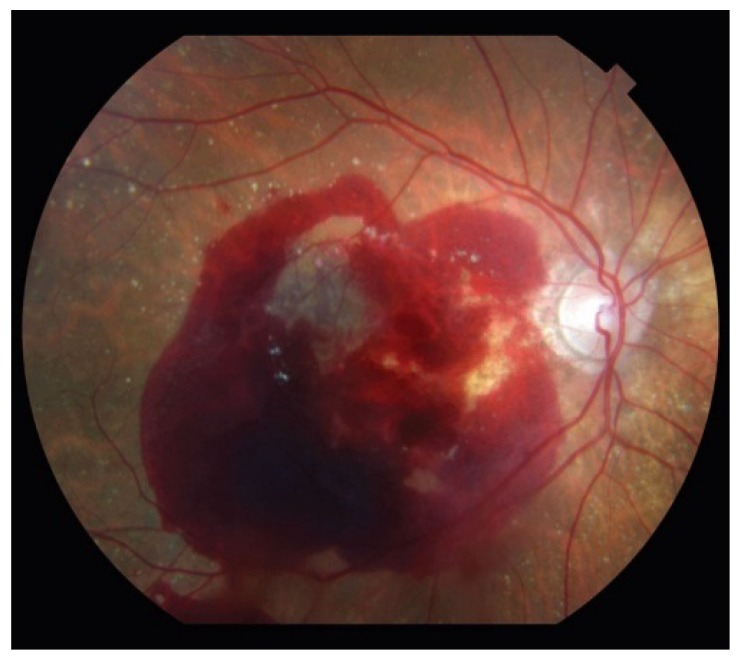
Neovascular age-related macular degeneration can also present with significant retinal hemorrhage.

**Figure 5:**
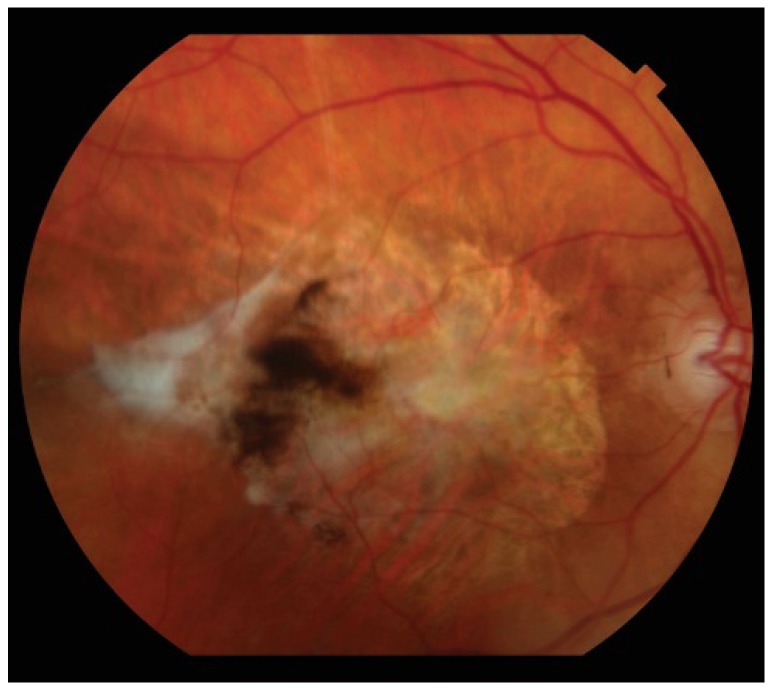
Subretinal fibrotic scarring is the end-stage manifestation of neovascular age-related macular degeneration.

CNV is the hallmark of neovascular AMD (Figure 3). “Type I” CNV involves the sub-RPE space and usually presents as a fibrovascular detachment of the RPE. “Type II” CNV involves the sub-neurosensory retinal space, and often appears as a gray-green lesion underneath the retina with overlying thickening of the retina. The endothelial cells of the pathologic new vasculature are incompetent, and result in fluid leakage and hemorrhage (Figure 4). The gold standard for diagnosing CNV is with fluorescein angiography, where CNV is seen as hyperfluorescent lesions deep in the retina that increase in intensity and size over time as the fluorescein leaks from the neovascular membranes. “Classic” CNV refers to well-demarcated lesions with early hyperfluorescence and clear leakage in later frames. “Occult” CNV is either a fibrovascular pigment epithelial detachment or ill-defined leakage from an undetermined source. The end result of CNV is a subretinal disciform scar, which often appears as a white/yellow lesion with variable degrees of pigmentation (Figure 5). 

## 6. Treatment of Non-Neovascular AMD

The Age Related Eye Disease Study (AREDS) was designed and funded by the National Eye Institute (NEI) of the National Institutes of Health (NIH) to determine whether antioxidant supplementation could have protective effects against AMD [7,38]. Some early epidemiological studies were promising, but others showed conflicting results [39,40,41,42,43,44]. AREDS was a multicenter, randomized, double-masked clinical trial, that enrolled 3640 participants and was stratified into four categories of severity: Category 1, total drusen area less than 5 small (<63 μm) drusen; Category 2, multiple small drusen or single/scattered intermediate drusen (63–124 μm), and/or pigmentary changes; Category 3, at least 1 large druse (≥125 μm), and/or extensive intermediate drusen, and/or geographic atrophy not involving the central macula; Category 4, advanced AMD or decreased vision caused by AMD in the fellow non-study eye. Participants were randomized to one of four arms: oral supplementation of antioxidants, zinc 80 mg, antioxidants + zinc, or placebo. The antioxidant regimen comprised vitamin C 500 mg, vitamin E 400 IU, and beta-carotene 15 mg. The zinc formulation contained copper 2 mg to prevent zinc-induced anemia. The placebo group provided an excellent understanding of AMD’s natural history. In Category 2, 1.3% of participants progressed to advanced AMD in 5 years, compared to 18% of Category 3, and 43% of Category 4. Of the treatment arms, the largest risk reduction was seen in Category 3 and 4 participants who received both antioxidants and zinc (odds radio (OR), 0.66 (34% odds reduction); *p* = 0.001). The risk reduction of losing 15 letters or more was an OR of 0.73 (27% odds reduction; *p* = 0.008).

The second AREDS study (AREDS2) was initiated in 2006 [45], to investigate whether omega-3 fatty acids (docosahexaenoic acid (DHA) and its precursor eicosapentaenoic acid (EPA)) and/or lutein and zeaxanthin would further slow the progression of AMD if added to the original AREDS formulation. Four thousand two hundred and three participants with bilateral large drusen, or large drusen in one eye and advanced AMD in the fellow eye, were randomized to one of four arms: (1) original AREDS formulation only; (2) original AREDS + lutein 10 mg + zeaxanthin 2 mg; (3) original AREDS + DHA 350 mg + EPA 650 mg; and (4) original AREDS + DHA/EPA + lutein/zeaxanthin. Participants were randomized in a secondary randomization to the original AREDS regimen with or without beta-carotene, and standard or lower doses of zinc. After a median of 5 years of follow-up, there was no added benefit in adding DHA/EPA or lutein/zeaxanthin. However, those randomized to the original AREDS formulation without beta-carotene but with lutein and zeaxanthin had slightly lower rates of progression (hazard ratio (HR) 0.82; *p* = 0.02). It was also noted that participants who were former smokers who took formulations with beta-carotene had higher likelihood of developing lung cancer (2.0% *vs.* 0.9%; *p* = 0.04). The updated suggestion was to remove beta-carotene, and include lutein/zeaxanthin, in the new formulation.

Many novel treatments for non-neovascular AMD are currently in development. Genetic variation in the complement pathway has been repeatedly implicated as a major contributor to AMD pathogenesis, and several trials are evaluating the effect of complement inhibitors. The 18-month Phase II MAHALO study enrolled 129 patients with bilateral geographic atrophy and showed that monthly intravitreal injections of lampalizumab (anti-factor D Fab, Roche/Genentech) achieved a 20% reduction in the growth rate of atrophy [46]. Interestingly, those with a particular complement factor I genetic variant benefited from a 44% reduction. Other promising concepts being explored as therapeutic strategies include methods that target inflammasomes [47], the visual pigment cycle in photoreceptors [48,49], neuroprotection [1,50,51,52,53], and stem cell transplantation [54,55].

## 7. Treatment of Neovascular AMD

One of the most significant recent advances in ophthalmology has been in the treatment of neovascular AMD. Laser photocoagulation was the first effective treatment, and its efficacy was shown in the Macular Photocoagulation Studies (MPS) that were initiated in the 1970s. Dramatic visual benefit was seen in extrafoveal lesions [56,57], and less so in parafoveal lesions [58,59]. Subfoveal lesions benefited from laser as well, but the practice of lasering the fovea was not ideal [60,61], and photodynamic therapy (PDT) was developed for treating choroidal neovascular membranes.

PDT uses photosensitizing agents that are administered intravenously and activated by photons at specific wavelengths. Free radical generation causes photochemical damage to the targeted tissue. Verteporfin is the FDA-approved photosensitizer for ophthalmic use, and it involves an intravenous infusion over 10 min. at a dose of 6 mg/m^2^ of body surface area. Laser light at 689 nm is applied 15 min. after the infusion to the area of CNV. The photosensitizer accumulates in the CNV, and PDT is able to target the lesion while sparing surrounding tissues. A phase III trial randomized 609 participants with subfoveal neovascular AMD to PDT or placebo, and showed that more eyes treated with PDT were spared from moderate vision loss (loss of <15 letters of visual acuity at 12 months; 61% *vs.* 46%; *p* < 0.001) [62]. Eyes with predominantly classic lesions on fluorescein angiography benefited the most (67% *vs.* 39%; *p* < 0.001). The effects were sustained at two years [63] and three years [64]. 

PDT was the first treatment to produce a significant decrease in the rates of visual loss from subfoveal neovascular AMD, and still has a role in AMD treatment today. However, it was not until the current anti-vascular endothelial growth factor (VEGF) era that patients routinely experienced improved vision. Pegaptanib (Macugen; Valeant Ophthalmics, Bridgewater, NJ, USA) was the first anti-VEGF agent that was FDA-approved for neovascular AMD. It is an RNA aptamer that binds VEGF_165_. The VISION studies randomized 1186 participants with subfoveal CNV to sham, 0.3 mg, 1.0 mg, or 3.0 mg pegaptanib intravitreal injections every six weeks [65]. The 0.3 mg group had 70% losing <15 letters, compared to 55% of the sham group (*p* < 0.001) at 48 weeks. Currently, newer more potent anti-VEGF agents have largely replaced pegaptanib.

Ranibizumab (Lucentis; Genentech, South San Francisco, California, CA, USA) is a monoclonal Fab fragment against all active VEGF-A isoforms. It was FDA-approved for the treatment of neovascular AMD in 2006 after its efficacy was demonstrated in the ANCHOR [66] and MARINA [67] trials. In the ANCHOR study, 430 participants with predominantly classic CNV were randomized to monthly 0.3 mg ranibizumab, 0.5 mg ranibizumab, or PDT, and loss of <15 letters at 12 months was seen in 94.3%, 96.4%, *vs.* 64.3%, respectively (*p* < 0.001). In fact, visual acuity improved by ≥15 letters in 35.7%, 40.3% *vs.* 5.6%, respectively (*p* < 0.001). In the MARINA trial, 716 participants with minimally classic or occult CNV were randomized to 0.3 mg ranibizumab, 0.5 mg ranibizumab, or sham injections. At 12 months, loss of <15 letters was seen in 94.5%, 94.6% *vs.* 62.2%, respectively (*p* < 0.001), and improvement by ≥15 letters in 24.8%, 33.8%, *vs.* 5.0%, (*p* < 0.001). Results from the pivotal ranibizumab trials are now the gold standard by which neovascular AMD treatments are evaluated (Figure 3).

Bevacizumab (Avastin; Genentech, South San Francisco, California, CA, USA), a monoclonal anti-VEGF-A antibody and the parent molecule of ranibizumab, was developed as a systemically administered chemotherapy, which is currently FDA-approved for colorectal cancer, non-small cell lung cancer, cervical cancer, glioblastoma, and renal cell carcinoma. However, many retina specialists use it off-label as a cost-effective alternative to ranibizumab for a multitude of retinal diseases. The NEI funded the CATT study to compare bevacizumab and ranibizumab for neovascular AMD [68,69]. One thousand two hundred and eight participants with neovascular AMD were randomized to ranibizumab or bevacizumab on either a monthly or “as-needed” schedule. Monthly bevacizumab and monthly ranibizumab had similar efficacies, and the results in the as-needed arms were also comparable. The disadvantages of intravitreal bevacizumab are the need to obtain the drug from compounding pharmacies and theoretical safety concerns due to the longer systemic half-life.

Aflibercept (Eylea; Regeneron, Tarrytown, New York, NY, USA) is the newest approved anti-VEGF agent, and is a decoy receptor that blocks all VEGF-A isoforms, VEGF-B, and placental growth factor (PlGF) [70,71]. It is a recombinant fusion protein made from ligand-binding elements of VEGF receptor 1 (VEGFR-1) and VEGFR-2, fused to the human IgG1 Fc segment. Aflibercept was approved by the FDA in 2011, and has provided an alternative method of VEGF suppression that was shown to have similar efficacy to ranibizumab when administered bimonthly rather than monthly. Aflibercept’s higher binding affinity is thought to be the main reason for its prolonged effects [72]. The VIEW trials were two parallel trials that randomized 2419 participants to 0.5 mg monthly aflibercept, 2.0 mg monthly aflibercept, 2.0 mg aflibercept every 2 months after 3 initial monthly doses, or monthly 0.5 mg ranibizumab [73]. All of the aflibercept groups, including the bimonthly group, were shown to be noninferior to monthly ranibizumab.

A new drug with promising early results is an inhibitor of platelet derived growth factor (PDGF). Pericytes are attracted to angiogenic endothelial cells in CNV by responding to PDGF signaling. They provide the endothelial cells with survival factors, including VEGF. E10030 (Fovista, Ophthotech, New York, NY, USA) is an anti-PDGF aptamer that binds PDGF and results in loss of pericytes from endothelial basement membranes, making the neovascular membranes more susceptible to anti-VEGF treatment. In a large Phase II trial, 449 participants with neovascular AMD were randomized to E10030 0.3 mg with ranibizumab 0.5 mg, E10030 1.5 mg with ranibizumab 0.5 mg, or sham with ranibizumab 0.5 mg. For the first time, a head-to-head treatment regimen was shown to be superior to ranibizumab monotherapy. The E10030 1.5 mg + ranibizumab group gained a mean of 10.6 ETDRS letters at 24 weeks, compared to 6.5 letters for ranibizumab monotherapy (*p* = 0.019) [74]. A Phase III study is currently enrolling patients. 

Given the multiple treatment options for neovascular AMD, the selection of medication and treatment modality depends on the type of lesion, systemic health, social circumstances, and economic considerations. Having multiple options has been particularly helpful in treating difficult cases. For example, switching from one anti-VEGF medication to another has been shown to be effective in recurrent and refractory CNV [75,76,77,78]. Also, combination therapy of anti-VEGF treatment and PDT is particularly effective in treating in a variant of neovascular AMD called polypoidal choroidal vasculopathy [79,80]. 

Other investigative modalities include low-dose radiotherapy as an adjunctive treatment [81,82]; other novel mechanisms of VEGF blockade in early stages of clinical development, including sustained delivery platforms [83,84,85,86,87]; and therapeutics that target angiogenic pathways beyond VEGF [88,89,90,91,92,93,94]. Home-monitoring devices for early detection of CNV development may also allow treatment to be initiated earlier for improved outcomes [95]. Studies suggest that earlier diagnosis of AMD in general may also be possible with devices that measure impaired dark adaptation [96]. 

## 8. Conclusions

Our current understanding of the pathogenesis of AMD remains imprecise. Genetic studies performed over the past decade have pointed toward complement-mediated inflammation and oxidative stress as key pathways in disease initiation. The pathological angiogenesis that is the hallmark of one subtype of advanced disease has long been recognized, and many of the landmark advances in ophthalmology in recent decades have been in the treatment of neovascular AMD. Patients who routinely became blind are now able to sustain or improve their vision with anti-VEGF therapy. Amongst photodynamic therapy, focal laser photocoagulation, pegaptanib, bevacizumab, ranibizumab and aflibercept, we now have multiple modalities and treatment options to treat choroidal neovascularization in the most effective manner. However, frequent intravitreal injections are costly and inconvenient for the patient. New treatment paradigms in development are focused on novel strategies for drug delivery or novel molecules that will reduce treatment burden, as well as targets beyond angiogenesis that may result in even better visual outcomes. The treatment of non-neovascular AMD is still an unmet need with tremendous potential impact. Effective strategies for halting and reversing the neurodegeneration that is the prominent feature of dry AMD are eagerly anticipated. 
